# Brief report: Cannabis and opioid use disorder among heart failure admissions, 2008–2018

**DOI:** 10.1371/journal.pone.0255514

**Published:** 2021-09-30

**Authors:** Fouad Chouairi, Clancy W. Mullan, Neal Ravindra, Katherine A. A. Clark, Edward M. Jaffe, Jasjit Bhinder, Michael Fuery, Avirup Guha, Tariq Ahmad, Nihar R. Desai

**Affiliations:** 1 Department of Internal Medicine, Duke University Medical Center, Durham, NC, United States of America; 2 Division of Cardiac Surgery, Yale School of Medicine, New Haven, CT, United States of America; 3 Division of Cardiovascular Medicine, Department of Internal Medicine, Yale School of Medicine, New Haven, CT, United States of America; 4 Department of Computer Science, Stanford University, Palo Alto, CA, United States of America; 5 Department of Anesthesiology and Critical Care Medicine, George Washington University, Washington, D.C., United States of America; 6 Case Western Reserve University, Cleveland, OH, United States of America; Maastricht University Medical Center, NETHERLANDS

## Abstract

**Background:**

In the United States, both cannabis use disorder (CUD) and opioid use disorder (OUD) have increased in prevalence. The prevalence, demographics, and costs of CUD and OUD are not well known in heart failure (HF) admissions. This study aimed to use a national database to examine the prevalence, demographics, and costs associated with CUD and OUD in HF.

**Methods:**

This study used the National Inpatient Sample from 2008 to 2018 to identify all primary HF admissions with and without the co-diagnosis of OUD or CUD using International Classification for Diagnosis, diagnosis codes. Demographics, costs, and trends were examined.

**Results:**

Between 2008 and 2018, we identified 11,692,995 admissions for HF of which 84,796 (0.8%) had a co-diagnosis of CUD only, and 67,137 (0.6%) had a co-diagnosis of OUD only. The proportion of HF admissions with CUD significantly increased from 0.3% in 2008 to 1.3% in 2018 (p<0.001). The proportion of HF admissions with OUD significantly increased from 0.2% in 2008 to 1.1% in 2018 (p<0.001). Patients admitted with HF and either CUD or OUD were younger, more likely to be Black, and from lower socioeconomic backgrounds (p<0.001, all). HF admissions with OUD or CUD had higher median costs compared to HF admissions without associated substance abuse diagnoses ($8,611 vs. $8,337 for CUD HF and $10,019 vs. $8,337 for OUD HF, p<0.001 for both).

**Conclusions:**

Among discharge records for HF, CUD and OUD are increasing in prevalence, significantly affect underserved populations and are associated with higher costs of stay. Future research is essential to better delineate the cause of these increased costs and create interventions, particularly in underserved populations.

## Introduction

Over the last 20 years the proportion of Americans struggling with substance abuse has increased dramatically [1]. Two of the most abused substances in the United States are cannabis and opioids. It is estimated that as many as 3.5 million Americans suffer from cannabis use disorder (CUD) and that more than 2.1 million Americans suffer from opioid use disorder (OUD) [2, 3].

Despite the growing prevalence of these diseases, little research has investigated their existence and effects among patients admitted for heart failure (HF). Institutional data has found that a significant proportion of HF patients suffer from CUD and OUD and that OUD is associated with an increased HF incidence rate [4]. However, there has been no investigation of the prevalence and effects of CUD and OUD in HF using generalizable data. This study aimed to use a nationally representative database to examine the prevalence, demographics, and costs associated with CUD and OUD in HF.

## Methods

### Data source

In this study, we performed a retrospective, cross-sectional analysis of the National Inpatient Sample (NIS) to identify discharge records with heart failure diagnoses between 2008 and 2018. The NIS is a publicly available, nationally representative 20% stratified, all-payer, claims-based inpatient discharge sampling database that is collected and maintained by the Agency of Healthcare Research and Quality (AHRQ) Healthcare Cost and Utilization Project (HCUP) and represents over 35 million annual hospitalizations in the United States. In 2012, the NIS underwent a redesign of its sampling strategy for which we accounted for by using the updated trend weight files to account for the weighting changes in 2012 [5]. All investigators with access to the NIS data have a signed data use agreement with HCUP.

### Study population and variables

We used *International Classification of Diseases*, *Ninth Revision*, *Clinical Modification*, (ICD-9-CM) and (ICD-10-CM) diagnosis codes to identify heart failure patients who were 18 years or older (**S1 Table**) [6]. We then utilized ICD-9-CM codes and ICD-10-CM codes previously validated in the psychiatry and cardiology literature to identify patients with cannabis use disorder and opioid use disorder (**S1 Table**) [4, 7, 8]. Patients with coding for CUD or OUD remission were excluded. Similarly, ICD 9-CM and ICD-10CM codes were used to identify the comorbidities in **Table 1** and this coding can be found in **S1 Table**.

**Table 1:** Demographics, outcomes, and costs of HF hospitalizations with and without CUD and OUD in the United States from 2008–2018.

Variables	No CUD or OUD	CUD Only	OUD Only	P-Value
	(N = 11,536,414)	(N = 84,796)	(N = 67,137)	
**Age, years; median [IQR]**	75.0 [63.0–84.0]	52.0 [42.0–58.0]	58.0 [51.0–67.0]	**<0.001**
**Female (%)**	49.3	21.6	40.0	**<0.001**
**Race (%)**				
White	67.0	34.7	51.7	**<0.001**
Black	20.2	53.2	35.0	**<0.001**
Hispanic	7.9	7.7	9.1	**<0.001**
Asian or Pacific Islander	2.0	1.5	0.8	**<0.001**
Native American	0.5	1.0	0.8	**<0.001**
Unknown	2.3	1.9	2.7	**<0.001**
**Comorbidities (%)**				
COPD	36.3	39.2	49.9	**<0.001**
CAD	55.0	41.1	44.3	**<0.001**
PVD	9.2	5.4	7.5	**<0.001**
DM	45.6	31.5	39.1	**<0.001**
Depression	9.2	12.1	18.2	**<0.001**
OSA	12.4	14.2	15.2	**<0.001**
Obesity	19.8	24.1	25.5	**<0.001**
**Hospital Type (%)**				
Rural	13.2	6.3	61.	**<0.001**
Urban Non-Teaching	34.6	23.9	26.2	**<0.001**
Urban Teaching	52.1	69.8	67.6	**<0.001**
**Hospital Region (%)**				
Northeast	19.8	11.4	23.2	**<0.001**
Midwest	23.2	22.0	19.3	**<0.001**
South	41.0	38.5	29.7	**<0.001**
West	16.0	28.0	27.8	**<0.001**
**Payer Information (%)**				
Medicare	74.4	29.0	48.9	**<0.001**
Medicaid	8.6	40.6	33.9	**<0.001**
Private Insurance	12.0	12.1	9.8	**<0.001**
Other	5.0	18.3	7.4	**<0.001**
Median Household Income* (%)				
Quartile 1 (Lowest)	33.2	50.9	43.6	**<0.001**
Quartile 2	26.6	23.4	23.6	**<0.001**
Quartile 3	22.5	17.3	20.1	**<0.001**
Quartile 4 (Highest)	17.7	8.4	12.7	**<0.001**
**Discharge Disposition (%)**				
Routine	51.0	75.6	57.5	**<0.001**
Transfer to Short Term Hospital	2.9	2.4	2.1	**<0.001**
Skilled Nursing Facility*	19.7	4.4	13.4	**<0.001**
Home Health Care	22.2	10.6	18.5	**<0.001**
Against Medical Advice	1.1	6.1	7.1	**<0.001**
Died in Hospital	3.0	0.8	1.3	**<0.001**
**Length of Stay, days; median [IQR]**	4[2–6]	3 [2–6]	4 [2–7]	**<0.001**
**Total Hospital Costs, 2020 USD; median [IQR]**	8337 [5071–14712]	8611 [5220–15148]	10019 [6014–17591]	**<0.001**

### Statistical methodology

HF admissions were separated into 4 cohorts: patients with neither CUD or OUD, patients with CUD only, and patients with OUD only. Patients with both CUD and OUD concurrently were excluded. Gender, race, hospital type, hospital region, insurance payer, comorbidities, and discharge disposition were compared between cohorts using chi-squared tests. Age, length of stay, and total cost of hospital stay were compared between cohorts using Kruskal-Wallis Tests. Kruskal-Wallis Tests were used due to tested non-normal distribution of continuous variables. Two-tailed tests were used for continuous variables. Hospital costs were calculated using the cost to charge ratios with local group averages provided by AHRQ and were adjusted for wage indexes and were standardized to 2020 dollars to account for inflation as per the department of labor [9]. The proportion of CUD and OUD among HF admissions was then trended over time and analyzed by linear regression. All statistical analyses were performed using SPSS v26 (IBM, Armonk, NY).

## Results

**Fig 1:**
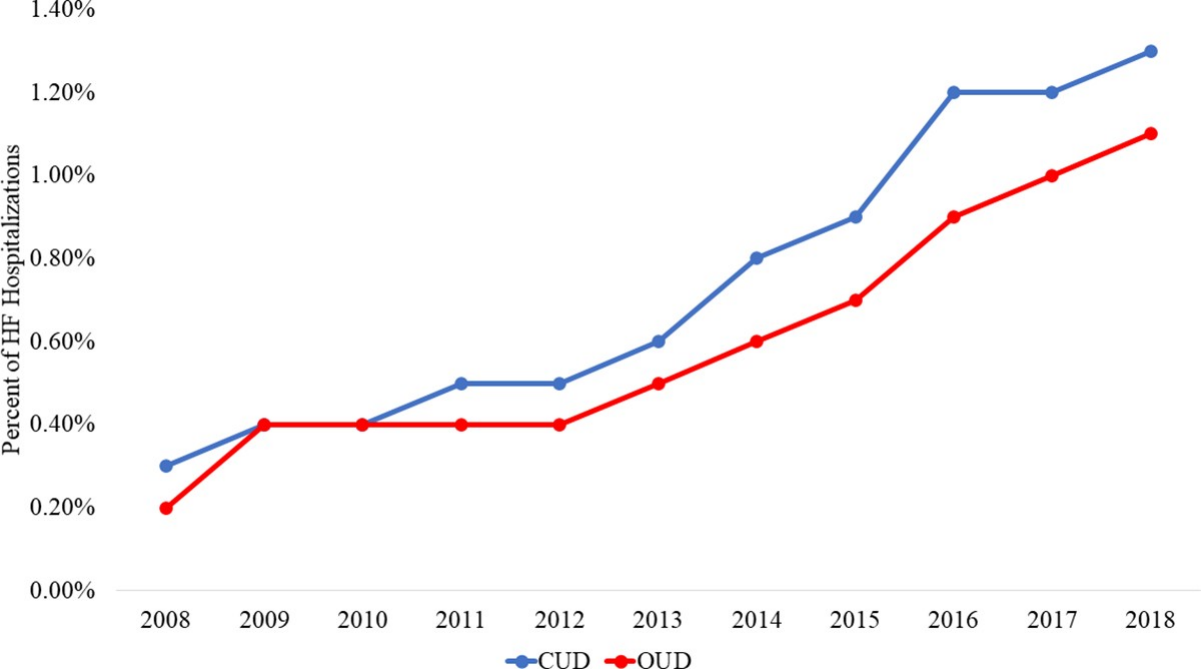
Percent of HF admissions with CUD and OUD over time: Primary HF admissions with a co-diagnosis of CUD or OUD were trended over 2008 to 2018 and then linear regression was used to analyze significance of trends. Linear regression: p<0.001, all.

Between 2008 and 2018, we identified 11,692,995 admissions for HF of which 84,796 (0.8%) had a co-diagnosis of CUD only, and 67,137 (0.6%) had a co-diagnosis of OUD only. Over time, the proportion of admissions with a history of CUD only increased from 0.3% in 2008 to 1.3% in 2018 (p<0.001). The proportion of admissions with a history of OUD only increased from 0.2% in 2008 to 1.1% in 2018 (p<0.001) (**Fig 1**). Hospital discharges of patients with CUD and OUD were significantly younger and had a higher proportion of Black race (CUD: 53.2%, OUD: 35.0% No CUD or OUD: 20.2%, p<0.001) and income in the first (lowest) quartile than records without a co-diagnosis CUD or OUD (CUD: 50.9% vs, OUD: 43.6%, No CUD or OUD: 33.2%, p<0.001). Both co-diagnoses are associated with increased proportions of hospitalizations with chronic obstructive pulmonary disease (COPD), obesity, obstructive sleep apnea (OSA), and depression (p<0.001, all). Additionally, CUD and OUD discharges were more likely to be treated at urban teaching hospitals, on the West coast, and to have been paid by Medicaid (**Table 1**).

In terms of discharge disposition, CUD hospitalizations had a higher proportion who were discharged home or discharged against medical advice. CUD admissions also had significantly higher median costs than patients with neither co-diagnosis ($8,611 vs. $8,337, p<0.001). Like CUD, OUD hospitalizations had a higher proportion who were discharged home or discharged against medical advice. OUD admissions also had significantly higher median costs than patients with neither co-diagnosis ($10,019 vs. $8,337, p<0.001) (**Table 1**).

## Discussion

In this national study of heart failure hospitalizations in the United States from 2008–2018, we found that both OUD and CUD more than tripled in prevalence. Significantly, the racial distributions of HF with and without CUD and OUD co-diagnoses differed, with a relative over-representation of Black race in the OUD and CUD populations compared to the HF population without co-diagnosis of OUD and CUD. Hospitalizations with OUD and CUD co-diagnoses also were more likely to be from the lowest income quartile and had higher rates of obesity, OSA, and depression. In HF hospitalizations, both CUD and OUD were associated with increased costs of hospitalization, and OUD admissions were nearly 20% more expensive.

We found that among HF hospitalizations, OUD prevalence has increased by a 5-fold while CUD has more than doubled. The current literature on HF patients with OUD is mixed. Some studies have found higher readmissions and hospitalization rates with OUD, while others have found no effects [4, 10]. Meanwhile, studies have described CUD as an independent predictor for the development of HF [11]. Additionally, it has also been theorized that due to marijuana’s effect of increasing plasma volume, it could increase the likelihood of HF exacerbation and a result hospitalization [12]. Given the increasing prevalence of substance abuse and an increase in cannabis legalization, it is important for future projects to investigate these outcomes on a national scale.

In addition to the possibility of more frequent admissions for these patients over time, we found that HF with co-diagnosis of CUD or OUD was associated with higher costs as compared to admissions without them. Over the last decades, the financial burden of HF on the healthcare system has increased dramatically [13]. To our knowledge there is no national study of the costs implications for co-diagnosis of OUD or CUD and HF. OUD HF admissions in particular incurred hospital costs of more than 19% greater than those without OUD.

We found that historically disenfranchised populations, particularly Black and lowest income quartile patients are over-represented in HF hospitalizations with OUD/CUD co-diagnosis relative to the hospitalizations without these co-diagnoses. A previous retrospective study at a single west coast hospital, found that patients with heart failure and substance abuse co-diagnosis were younger, more likely to be male, to be black, and to lack medical insurance [4]. They also found that these patient’s had higher proportions of ischemic heart disease, cerebrovascular accidents, and endocarditis than patients without substance abuse. These differences were largely attributed to socioeconomic barriers to care.

However, there may also be toxicologic mechanisms relevant to heart failure and the readmission in these patients. Cannabis use is an independent risk predictor of heart failure [11]. Mechanistically, this could be in part due to cannabinoid agonisms of the CB-1 receptor which has been shown to have several affects in humans including decreased myocardial contractility, endothelial dysfunction, tissue injury fibrosis and cell death [14, 15].

While methadone has a well characterized side effect of prolonged QT interval, the other opioids may not have as direct cardiotoxicity [16]. However the combination of CHF and opioid use is associated with increased likelihood of cardiovascular events [17]. CHF is highly associated with central and obstructive sleep apnea and CHF patients have weakened respiratory mechanics [18, 19]. The central respiratory depressing effect of opioids may explain increased morbidity with co-diagnosis of CHF and OUD [20].

Prior studies found that lower socioeconomic status populations were more likely to suffer from opioid overdose events [21] and that recent increases in cannabis use have been especially pronounced in Black and lower income communities [22]. CUD and OUD HF admissions also have significantly higher proportions of Medicaid payments and patients from the lowest income quartile. We recommend future studies with more granular, longitudinal follow up data to investigate HF outcomes in these populations. These investigations may be able to better elucidate compounding deleterious affects of HF with CUD and OUD.

### Limitations

Limitations of this study stem from the nature of the database and ICD codes, as there is a chance of misidentification or under-identification of hospitalizations due to code sensitivity. In addition to the possibility of frequent re-admission due to HF [4], among patients with a history of substance abuse, there is an increased likelihood of readmission so one patient with CUD or OUD could be readmitted multiple times having a disproportionate effect on the study [23]. As the NIS does not provide a patient-level identifier to track hospitalizations of a single patient over time, we are not able to account for this possibility of capture multiple times within the analysis. Finally, this study is observational and the variables are not longitudinal, limiting our ability to identify patient outcomes beyond their hospitalization.

## Conclusions

In conclusion, among hospitalizations for HF from 2008 to 2018 in the United States, the co-diagnoses of CUD and OUD are growing in prevalence, and underserved populations were over-represented within the OUD/CUD cohorts. These comorbidities were associated with higher costs of stay. It is important for future research to delineate the cause of these increased costs better and create potential interventions, particularly in underserved populations, to treat and subvert the negative effects of substance abuse among HF patients.

## Supporting information

S1 Table ICD-9-CM and ICD-10 codes for classification of heart failure, cannabis use disorder, and opioid use disorder. (DOCX)
