# Supplementary material for: Brief report: Cannabis and opioid use disorder among heart failure admissions, 2008–2018
Source: PLoS One. 2021 Sep 30;16(9):e0255514. doi: 10.1371/journal.pone.0255514 (PMC8483306; doi:10.1371/journal.pone.0255514)
Supplement: S1 Table — (DOCX) [file pone.0255514.s001.docx]

**S1 Table.** ICD-9-CM and ICD-10 codes for classification of heart failure, cannabis use disorder, and opioid use disorder

| **Diagnoses** | **ICD-9** | **ICD-10** |
| --- | --- | --- |
| Heart Failure | 398.91, 402.01, 402.11, 402.91, 404.01, 404.03, 404.11, 404.13, 404.91, 404.93, 428.x | I11.0, I13.0, I13.2, I09.81, I09.9, I25.5, I42.0, I425-I42.9, I43.x, I50.x |
| Cannabis Use Disorder (CUD) | '3043', '30430', '30431', '30432', '3052', '30520', '30521', '30522' | 'F1210', 'F12120', 'F12121', 'F12122', 'F12129', 'F1213', 'F12150', 'F12151', 'F12159', 'F12180', 'F12188', 'F1219', 'F1220', 'F1222', 'F12220', 'F12221', 'F12222', 'F12229', 'F1223', 'F1225', 'F12250', 'F12251', 'F12259', 'F1228', 'F12280', 'F12288', 'F1229', 'F129', 'F1290', 'F1292', 'F12920', 'F12921', 'F12922', 'F12929', 'F1293', 'F1295', 'F12950', 'F12951', 'F12959', 'F1298', 'F12980', 'F12988', 'F1299' |
| Opioid Use Disorder (OUD) | '3040', '30400', '30401', '30402', '3047', '30470', '30471', '30472', '3055', '30550', '30551', '30553', '9650', '96500', '96501', '96502', '96502', '96509' | 'F1110', 'F11120', 'F11121', 'F11122', 'F11129', 'F1114','F11150', 'F11151', 'F11159', 'F11181', 'F11182', 'F11188', 'F1119', 'F1120', 'F11220', 'F11221', 'F11222', 'F11229', 'F1123', 'F1124', 'F11250', 'F11251', 'F11259', 'F11281', 'F11282', 'F11288', 'F1129', 'F119', 'F1190', 'F1192', 'F11920', 'F11921', 'F11922', 'F11929', 'F1193', 'F1195', 'F11950', 'F11951', 'F11959', 'F1198', 'F11980', 'F11981', 'F11982', 'F11988', 'F1199' |
| COPD | '490', '4910', '4911', '4912', '49120', '49121', '49122', '4918', '4919', '4920', '4928', '494', '4940', '4941', '496', '49300', '49301', '49302', '49310', '49311', '49312', '49320', '49321', '49322', '49381', '49382', '49390', '49391', '49392', '4940', '4941', '4950', '4951', '4952', '4953', '4954', '4955', '4956', '4957', '4958', '4959', '500', '501', '502', '503', '504', '505', '5060', '5061', '5062', '5063', '5064', '5069', '5081', '5082', '5088', '5089' | 'I2781', 'I2782', 'I279', 'J40', 'J41', 'J410', 'J411', 'J418', 'J42', 'J43', 'J430', 'J431', 'J432', 'J438', 'J439', 'J44', 'J440', 'J441', 'J448', 'J449', 'J45', 'J452', 'J4520', 'J4521', 'J4522', 'J453', 'J4530', 'J4531', 'J4532', 'J454', 'J4540', 'J4541', 'J4542', 'J455', 'J4550', 'J4551', 'J4552', 'J459', 'J4590', 'J45901', 'J45902', 'J45909', 'J4599', 'J45990', 'J45991', 'J45998', 'J47', 'J470', 'J471', 'J479', 'J60', 'J61', 'J62', 'J620', 'J628', 'J63', 'J630', 'J631', 'J632', 'J633', 'J634', 'J635', 'J636', 'J64', 'J65', 'J66', 'J660', 'J661', 'J662', 'J668', 'J67', 'J670', 'J671', 'J672', 'J673', 'J674', 'J675', 'J676', 'J677', 'J678', 'J679', 'J684', 'J701', 'J703' |
| CAD | '4100', '41000', '41001', '41002', '4101', '41010', '41011', '41012', '4102', '41020', '41021', '41022', '4103', '41030', '41031', '41032', '4104', '41040', '41041', '41042', '4105', '41050', '41051', '41052', '4106', '41060', '41061', '41062', '4107', '41070', '41071', '41072', '4108', '41080', '41081', '41082', '4109', '41090', '41091', '41092', '4110', '4111', '4118', '41181', '41189', '412', '4130', '4131', '4139', '4140', '41400', '41401', '41402', '41403', '41404', '41405', '41406', '41407', '41410', '41411', '41412', '41419', '4142', '4143', '4144', '4148', '4149', 'V4581', 'V4582' | 'I20', 'I200', 'I201', 'I21', 'I210', 'I2101', 'I2102', 'I2019', 'I211', 'I2111', 'I2119', 'I212', 'I2121', 'I2129', 'I213', 'I214', 'I219', 'I22', 'I220', 'I221', 'I228', 'I229', 'I23', 'I230', 'I231', 'I232', 'I233', 'I234', 'I235', 'I236', 'I237', 'I238', 'I240', 'I241', 'I248', 'I249', 'I251', 'I2510', 'I2511', 'I25110', 'I25111', 'I25118', 'I25119', 'I252', 'I253', 'I254', 'I2541', 'I2542', 'I255', 'I256', 'I257', 'I2570', 'I25700', 'I25702', 'I25708', 'I25709', 'I2571', 'I25720', 'I25711', 'I25718', 'I25719', 'I2572', 'I25720', 'I25721', 'I25728', 'I25729', 'I2573', 'I25730', 'I25731', 'I25738', 'I25739', 'I2575', 'I25750', 'I25751', 'I25758', 'I25759', 'I2576', 'I25760', 'I25761', 'I25768', 'I25769', 'I2579', 'I25790', 'I25791', 'I25798', 'I25799', 'I258', 'I2581', 'I25810', 'I25811', 'I25812', 'I2582', 'I2583', 'I2584', 'I2589', 'I259' |
| PVD | '4400', '4401', '4402', '44020', '44021', '44022', '44023', '44029', '4404', '4408', '4409', '4439', '5570', '5571', '5579', '4413', '4414', '4415', '4416', '4417', '4419', '4420', '4421', '4422', '4423', '44281', '44282', '44283', '44284', '44289', '4429', '44321', '44322', '44323', '44324', '44329', '44770', '44771', '44772', '44773', '7854', '4431', '4439' | 'I70', 'I700', 'I701', 'I702', 'I708', 'I709', 'I71', 'I710', 'I711', 'I712', 'I713', 'I714', 'I715', 'I716', 'I718', 'I719', 'I731', 'I738', 'I739', 'I771', 'I790', 'I792', 'K551', 'K558', 'K559', 'Z958', 'Z959') \| ANY (I10_DX2, 'I70', 'I700', 'I701', 'I702', 'I708', 'I709', 'I71', 'I710', 'I711', 'I712', 'I713', 'I714', 'I715', 'I716', 'I718', 'I719', 'I731', 'I738', 'I739', 'I771', 'I790', 'I792', 'K551', 'K558', 'K559', 'Z958', 'Z959' |
| DM | '250', '2500', '25000', '25001', '25002', '25003', '2501', '25010', '25011', '25012', '25013', '2502', '25020', '25021', '25022', '25023', '2503', '25030', '25031', '25032', '25033', '2504', '25040', '25041', '25042', '25043', '2505', '25050', '25051', '25052', '25053', '2506', '25060', '25061', '25062', '25063', '25070', '25071', '25072', '25073', '25080', '25081', '25082', '25083', '25090', '25091', '25092', '25093' | 'E10', 'E100', 'E1000', 'E1001', 'E101', 'E1010', 'E1011', 'E102', 'E1021', 'E1022', 'E1029', 'E103', 'E1031', 'E10311', 'E10319', 'E1032', 'E10321', 'E103211', 'E103212', 'E103213', 'E103219', 'E10329', 'E103291', 'E103292', 'E103293', 'E103299', 'E1033', 'E10331', 'E103311', 'E103312', 'E103313', 'E103319', 'E10339', 'E103391', 'E103392', 'E103393', 'E103399', 'E1034', 'E10341', 'E103411', 'E103412', 'E103413', 'E103419', 'E10349', 'E103491', 'E103492', 'E103493', 'E103499', 'E1035', 'E10351', 'E103511', 'E103512', 'E103513', 'E103519', 'E10352', 'E103521', 'E103522', 'E103523', 'E103529', 'E10353', 'E103531', 'E103532', 'E103533', 'E103539', 'E10354', 'E103541', 'E103542', 'E103543', 'E103549', 'E10355', 'E103551', 'E103552', 'E103553', 'E103559', 'E10359', 'E103591', 'E103592', 'E103593', 'E103599', 'E1036', 'E1037', 'E1037X1', 'E1037X2', 'E1037X3', 'E1037X9', 'E1039', 'E104', 'E1040', 'E1041', 'E1042', 'E1043', 'E1044', 'E1049', 'E105', 'E1051', 'E1052', 'E1059', 'E106', 'E1061', 'E10610', 'E10618', 'E1062', 'E10620', 'E10621', 'E10622', 'E10628', 'E1063', 'E10630', 'E10638', 'E1064', 'E10641', 'E10649', 'E1065', 'E1069', 'E108', 'E109',  'E11', 'E110', 'E1100', 'E1101', 'E111', 'E1110', 'E1111', 'E112', 'E1121', 'E1122', 'E1129', 'E113', 'E1131', 'E11311', 'E11319', 'E1132', 'E11321', 'E113211', 'E113212', 'E113213', 'E113219', 'E11329', 'E113291', 'E113292', 'E113293', 'E113299', 'E1133', 'E11331', 'E113311', 'E113312', 'E113313', 'E113319', 'E11339', 'E113391', 'E113392', 'E113393', 'E113399', 'E1134', 'E11341', 'E113411', 'E113412', 'E113413', 'E113419', 'E11349', 'E113491', 'E113492', 'E113493', 'E113499', 'E1135', 'E11351', 'E113511', 'E113512', 'E113513', 'E113519', 'E11352', 'E113521', 'E113522', 'E113523', 'E113529', 'E11353', 'E113531', 'E113532', 'E113533', 'E113539', 'E11354', 'E113541', 'E113542', 'E113543', 'E113549', 'E11355', 'E113551', 'E113552', 'E113553', 'E113559', 'E11359', 'E113591', 'E113592', 'E113593', 'E113599', 'E1136', 'E1137', 'E1137X1', 'E1137X2', 'E1137X3', 'E1137X9', 'E1139', 'E114', 'E1140', 'E1141', 'E1142', 'E1143', 'E1144', 'E1149', 'E115', 'E1151', 'E1152', 'E1159', 'E116', 'E1161', 'E11610', 'E11618', 'E1162', 'E11620', 'E11621', 'E11622', 'E11628', 'E1163', 'E11630', 'E11638', 'E1164', 'E11641', 'E11649', 'E1165', 'E1169', 'E118', 'E119',  'E12', 'E120', 'E1200', 'E1201', 'E121', 'E1210', 'E1211', 'E122', 'E1221', 'E1222', 'E1229', 'E123', 'E1231', 'E12311', 'E12319', 'E1232', 'E12321', 'E123211', 'E123212', 'E123213', 'E123219', 'E12329', 'E123291', 'E123292', 'E123293', 'E123299', 'E1233', 'E12331', 'E123311', 'E123312', 'E123313', 'E123319', 'E12339', 'E123391', 'E123392', 'E123393', 'E123399', 'E1234', 'E12341', 'E123411', 'E123412', 'E123413', 'E123419', 'E12349', 'E123491', 'E123492', 'E123493', 'E123499', 'E1235', 'E12351', 'E123511', 'E123512', 'E123513', 'E123519', 'E12352', 'E123521', 'E123522', 'E123523', 'E123529', 'E12353', 'E123531', 'E123532', 'E123533', 'E123539', 'E12354', 'E123541', 'E123542', 'E123543', 'E123549', 'E12355', 'E123551', 'E123552', 'E123553', 'E123559', 'E12359', 'E123591', 'E123592', 'E123593', 'E123599', 'E1236', 'E1237', 'E1237X1', 'E1237X2', 'E1237X3', 'E1237X9', 'E1239', 'E124', 'E1240', 'E1241', 'E1242', 'E1243', 'E1244', 'E1249', 'E125', 'E1251', 'E1252', 'E1259', 'E126', 'E1261', 'E12610', 'E12618', 'E1262', 'E12620', 'E12621', 'E12622', 'E12628', 'E1263', 'E12630', 'E12638', 'E1264', 'E12641', 'E12649', 'E1265', 'E1269', 'E128', 'E129',  'E13', 'E130', 'E1300', 'E1301', 'E131', 'E1310', 'E1311', 'E132', 'E1321', 'E1322', 'E1329', 'E133', 'E1331', 'E13311', 'E13319', 'E1332', 'E13321', 'E133211', 'E133212', 'E133213', 'E133219', 'E13329', 'E133291', 'E133292', 'E133293', 'E133299', 'E1333', 'E13331', 'E133311', 'E133312', 'E133313', 'E133319', 'E13339', 'E133391', 'E133392', 'E133393', 'E133399', 'E1334', 'E13341', 'E133411', 'E133412', 'E133413', 'E133419', 'E13349', 'E133491', 'E133492', 'E133493', 'E133499', 'E1335', 'E13351', 'E133511', 'E133512', 'E133513', 'E133519', 'E13352', 'E133521', 'E133522', 'E133523', 'E133529', 'E13353', 'E133531', 'E133532', 'E133533', 'E133539', 'E13354', 'E133541', 'E133542', 'E133543', 'E133549', 'E13355', 'E133551', 'E133552', 'E133553', 'E133559', 'E13359', 'E133591', 'E133592', 'E133593', 'E133599', 'E1336', 'E1337', 'E1337X1', 'E1337X2', 'E1337X3', 'E1337X9', 'E1339', 'E134', 'E1340', 'E1341', 'E1342', 'E1343', 'E1344', 'E1349', 'E135', 'E1351', 'E1352', 'E1359', 'E136', 'E1361', 'E13610', 'E13618', 'E1362', 'E13620', 'E13621', 'E13622', 'E13628', 'E1363', 'E13630', 'E13638', 'E1364', 'E13641', 'E13649', 'E1365', 'E1369', 'E138', 'E139',  'E14', 'E140', 'E1400', 'E1401', 'E141', 'E1410', 'E1411', 'E142', 'E1421', 'E1422', 'E1429', 'E143', 'E1431', 'E14311', 'E14319', 'E1432', 'E14321', 'E143211', 'E143212', 'E143213', 'E143219', 'E14329', 'E143291', 'E143292', 'E143293', 'E143299', 'E1433', 'E14331', 'E143311', 'E143312', 'E143313', 'E143319', 'E14339', 'E143391', 'E143392', 'E143393', 'E143399', 'E1434', 'E14341', 'E143411', 'E143412', 'E143413', 'E143419', 'E14349', 'E143491', 'E143492', 'E143493', 'E143499', 'E1435', 'E14351', 'E143511', 'E143512', 'E143513', 'E143519', 'E14352', 'E143521', 'E143522', 'E143523', 'E143529', 'E14353', 'E143531', 'E143532', 'E143533', 'E143539', 'E14354', 'E143541', 'E143542', 'E143543', 'E143549', 'E14355', 'E143551', 'E143552', 'E143553', 'E143559', 'E14359', 'E143591', 'E143592', 'E143593', 'E143599', 'E1436', 'E1437', 'E1437X1', 'E1437X2', 'E1437X3', 'E1437X9', 'E1439', 'E144', 'E1440', 'E1441', 'E1442', 'E1443', 'E1444', 'E1449', 'E145', 'E1451', 'E1452', 'E1459', 'E146', 'E1461', 'E14610', 'E14618', 'E1462', 'E14620', 'E14621', 'E14622', 'E14628', 'E1463', 'E14630', 'E14638', 'E1464', 'E14641', 'E14649', 'E1465', 'E1469', 'E148', 'E149' |
| Depression | '2962', '29620', '29621', '29622', '29623', '29624', '29625', '2963', '29630', '29631', '29632', '29633', '29634', '29635', '3004', '311', '2965', '29650', '29651', '29652', '29653', '2966', '29660', '29661', '29662', '29663', '29682', '29690', '3090', '3091', '30928' | 'F32', 'F320', 'F321', 'F322', 'F323', 'F324', 'F325', 'F328', 'F3281', 'F3289', 'F329', 'F33', 'F330', 'F331', 'F332', 'F3338', 'F339', 'F341', 'F342', 'F313', 'F3130', 'F3131', 'F3132', 'F314', 'F315', 'F316', 'F3160', 'F3161', 'F3162', 'F3163', 'F3164', 'F348', 'F3481', 'F3489', 'F349', 'F380', 'F381', 'F388', 'F39', 'F99' |
| OSA | '32723' | 'G4733' |
| Obesity | '27800', '27801', '27803', 'V8530', 'V8531', 'V8532', 'V8533', 'V8534', 'V8535', 'V8536', 'V8537', 'V8538', 'V8539', 'V8540', 'V8541', 'V8542', 'V8543', 'V8544', 'V8545' | 'E661', 'E662', 'E668', 'E669', 'E6601', 'Z6830', 'Z6831', 'Z6832', 'Z6833', 'Z6834', 'Z6835', 'Z6836', 'Z6837', 'Z6838', 'Z6839', 'Z6840', 'Z6841', 'Z6842', 'Z6843', 'Z6844', 'Z6845') \| ANY (I10_DX2, 'E661', 'E662', 'E668', 'E669', 'E6601', 'Z6830', 'Z6831', 'Z6832', 'Z6833', 'Z6834', 'Z6835', 'Z6836', 'Z6837', 'Z6838', 'Z6839', 'Z6840', 'Z6841', 'Z6842', 'Z6843', 'Z6844', 'Z6845' |
